# Cerebellar degeneration in primary Sjögren syndrome: a case report

**DOI:** 10.1186/s13256-021-03103-x

**Published:** 2021-10-19

**Authors:** Seow Lin Chuah, Ahmad Tirmizi Jobli, Sharifah Aishah Wan, Cheng Lay Teh

**Affiliations:** 1 Rheumatology Unit, Medical Department, Sarawak General Hospital, Kuching, Malaysia; 2 Radiology Department, Faculty of Medicine and Health Sciences, University Malaysia Sarawak, Kota Samarahan, Malaysia

**Keywords:** Sjögren syndrome, Ataxia, Cerebellar atrophy, Cyclophosphamide

## Abstract

**Background:**

Cerebellar degeneration is a rare and severe presentation of primary Sjögren syndrome. There are few case reports of cerebellar degeneration associated with different autoimmune diseases, especially with systemic lupus erythematosus and neuro-Behcet’s disease. There are only six patients reported worldwide to be affected by cerebellar atrophy associated with primary Sjögren syndrome. In this report, we describe a patient with primary Sjögren syndrome who presented with ataxia due to cerebellar degeneration.

**Case presentation:**

We report the case of a 37-year-old Chinese woman with primary Sjögren syndrome who presented with ataxia over 3 months associated with tremor of the limbs. Magnetic resonance imaging of the brain revealed bilateral cerebellar atrophy. Based on the presence of cerebellar signs with magnetic resonance imaging brain findings, she was diagnosed as cerebellar degeneration secondary to primary Sjögren syndrome. She was treated with methylprednisolone, hydroxychloroquine, and two cycles of monthly intravenous cyclophosphamide. Subsequently, she refused further treatment, and her neurological symptoms remained the same upon the last clinic review. Primary cerebellar degeneration is rarely associated with primary Sjögren syndrome. The pathogenesis of the neurological manifestations in primary Sjögren syndrome is unclear. Treatment involves corticosteroids and immunosuppressive agents with no consensus of a specific therapy for the management of primary Sjögren syndrome with central nervous system involvement.

**Conclusions:**

Cerebellar degeneration is a rare presentation of primary Sjögren syndrome. Early diagnosis and treatment of this condition is needed to ensure a good outcome.

## Background

Primary Sjögren syndrome (PSS) is an autoimmune connective tissue disease, characterized by mononuclear infiltration and destruction of salivary and lacrimal glands leading to xerostomia and xerophthalmia. It predominantly affects female patients between 40 and 50 years of age [1].

SS may occur as primary or secondary to another connective tissue disease (mainly systemic lupus erythematosus, rheumatoid arthritis, or scleroderma). Approximately 35% of patients with primary Sjögren syndrome (PSS) suffer from systemic manifestations [2]. Neurological disorders are one of the extraglandular manifestations of the disease. In 25–60% of cases, the neurological symptoms preceded the diagnosis of PSS by 2 years. In the remaining patients, neurological disorders appeared 6–8 years after diagnosis [3].

The prevalence of neurological manifestations in PSS varies widely from 10% to 60% [4–6]. The most common neurological complication of PSS is peripheral neuropathy, particularly sensory polyneuropathy [7]. Central nervous system (CNS) involvement is much less common (2–25%) [8, 9]. Although ataxia due to PSS has also been described, marked cerebellar atrophy associated with PSS has rarely been reported [10, 11]. There are few reports of cerebellar degeneration associated with different autoimmune diseases, especially with SLE [12, 13] and neuro-Behcet’s disease [14, 15]. In this case report, we describe a patient with PSS who presented with ataxia due to cerebellar degeneration.

This case highlights the challenges in the evaluation of a patient who presents with cerebellar signs. There is a wide range of potential causes that need to be considered and excluded. We also performed a literature review of previously published cases.

## Case presentation

A 37-year-old Chinese female with a history of primary Sjögren syndrome presented with progressive unsteady gait for 3 months. She first presented with inflammatory arthritis involving bilateral shoulders, wrists, hands, knees, and feet in 2014. She also had sicca syndrome, and she tested positive for Schirmer’s test in both eyes. Her anti-SSA was positive. Laboratory tests were positive for speckled-type antinuclear antibodies and rheumatoid factor. Anti-double-stranded deoxyribonucleic acid (anti dsDNA) antibody test was negative. Assay for antiphospholipid antibodies (anticardiolipin and anti-*B*2 glycoprotein), SS-B/anti-La, human immunodeficiency virus (HIV) antibody, hepatitis B surface antigen (Hbs antigen), and hepatitis C virus (HCV) antibody were all negative. She was diagnosed as PSS and treated with prednisolone, hydroxychloroquine, and methotrexate. One year later, she developed bilateral lower limb numbness and a nerve conduction study showed evidence of mononeuritis multiplex involving bilateral tibial and peroneal nerves. She was treated with intravenous methylprednisolone leading to full neurological recovery later.

In August 2016, she presented with bruises, thrombocytopenia (platelets 5 × 10^3^/µL) and leukopenia (total white cells (TWC): 3.3 × 10^3^/µL). She underwent further workup, and bone marrow aspiration and trephine (BMAT) showed increased megakaryopoiesis, which was suggestive of peripheral destruction of platelet. B-lymphoproliferative studies showed no evidence of clonal B-lymphoproliferative disorder. Computed tomography (CT) of the neck, thorax, abdomen, and pelvis revealed multiple prominent intraparotid, cervical, left supraclavicular, and bilateral axillary lymph nodes; posterior nasopharynx diffuse bulkiness was likely due to lymphoid tissue. Fine needle aspiration cytology (FNAC) of posterior nasal space demonstrated scattered lymphoid cells likely due to florid reactive lymphoid proliferation. She was treated with intravenous immunoglobulin (IVIG) 1 mg/kg/day and followed by prednisolone 70 mg with gradual tapering dose. Her blood counts normalized subsequently. In 2017, she was treated for herpes zoster infection.

In January 2018, she had palpable cervical lymph nodes. CT scan reported multiple prominent lymph nodes in the neck and diffuse enlargement of the parotid glands. The large right-sided intraparotid mass was markedly reduced. The axillary lymph nodes noted previously had also reduced. However, interval development of multiple pulmonary nodules with areas of consolidation were seen. There were stable cystic changes in the lungs. Biopsy of the posterior nasal space showed atypical lymphoid infiltrate. Flow cytometry of the posterior nasal space reported intermediate-size, kappa-restricted B-cells along with increased plasma cells, suggestive of marginal zone lymphoma. PET scan revealed fluorodeoxyglucose (FDG)-avid bilateral cervical, and right intraparotid nodes were suspicious for disease. Bilateral multiple minimal to intensely FDG-avid pulmonary nodules of varying sizes were seen. Subsequently, she refused further interventions and was discharged with hydroxychloroquine 200 mg, prednisolone 7.5 mg, and calcium carbonate 1 g daily.

There was history of nonadherence to medical therapy since 2014. She was not under rheumatology follow-up since March 2018. She only took 5 mg of prednisolone, 500 mg of calcium carbonate, and thyroxine 100 μg daily from her general practitioner after she was noted to have hypothyroidism.

She presented to us in 2019 with 3 months history of unsteady gait and tremors over all four limbs. There was no family history of gait disturbance or familial history of neurological disorders. The patient had no exposure to toxins such as alcohol. She denied smoking and recreational drug usage. She was married with no children. She lost her job as a clerk since she acquired unsteady gait. There was no known food intolerance, medication allergy, or significant past surgical history.

On examination, the patient was afebrile and normotensive with a pulse rate of 80 beats per minute. Respiratory, cardiovascular, and abdominal examinations were normal. Her neurological examination revealed ataxia. She was unable to walk without assistance. She also had dysmetria, dysarthria, and intention tremor of the four limbs, especially her hands. Other motor and sensory examinations were normal. Her deep tendon reflexes were normoactive, and her plantar responses were flexor bilaterally. She had horizontal nystagmus and titubation. Examination of the cranial nerves did not reveal any abnormalities. There was Jaccoud deformity of both hands with limited range of movement of the right wrist. She did not have active synovitis. Left parotid swelling was present. There was no lymphadenopathy.

## Investigations

**Fig. 1:**
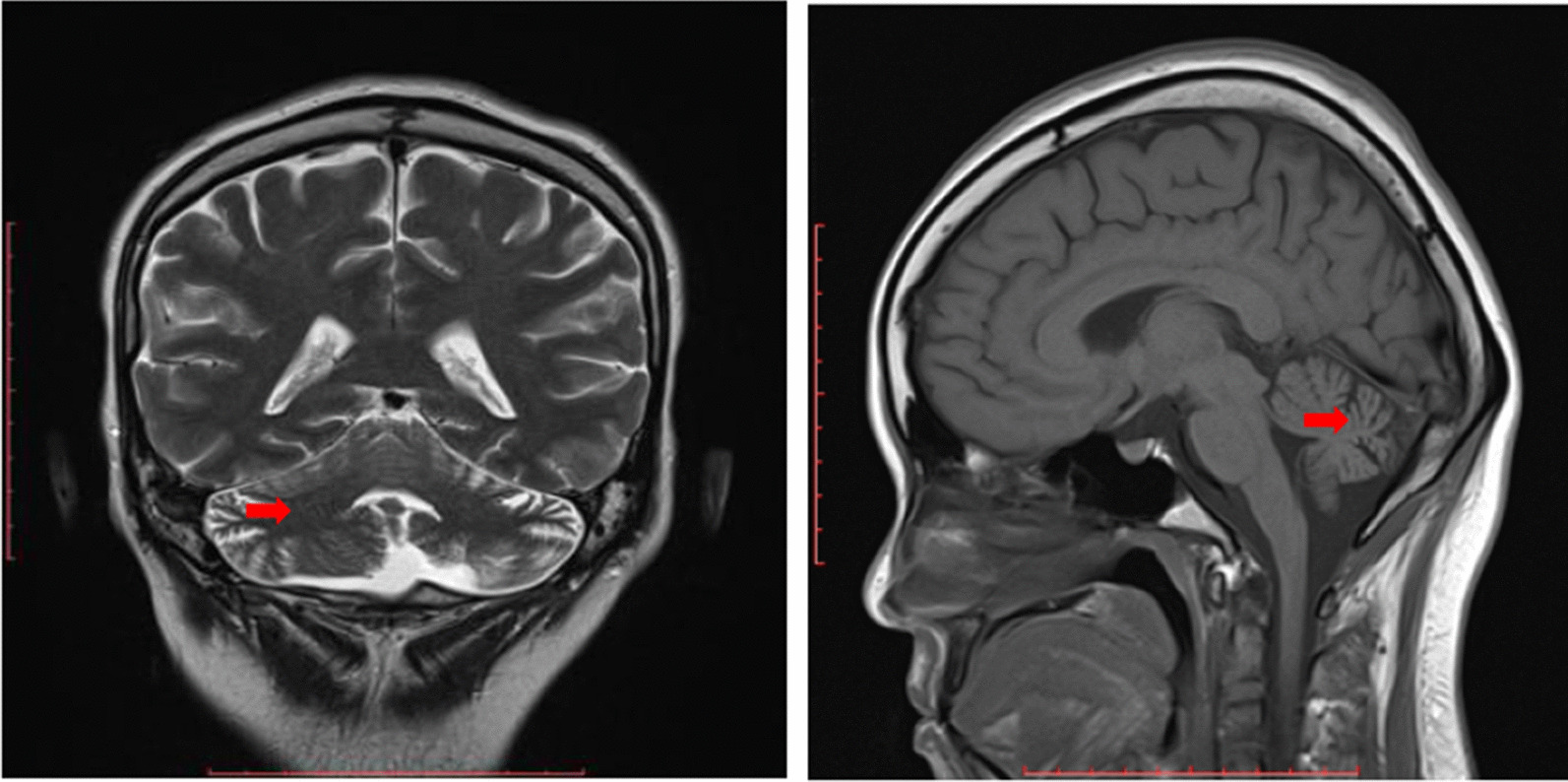
Coronal T2WI (left) and sagittal T1WI (right) showing atrophy of the superior and inferior cerebellar vermis (arrow)

Laboratory investigations reveal hemoglobin of 11.8 g/dL, white blood cells of 5.08 × 10^3^/μL, and platelet counts of 285 × 10^3^/μL. Her renal profile revealed creatinine level of 60 mmol/L with no electrolyte imbalance. Liver function test showed slightly low albumin level at 33 g/L with normal liver enzymes. Urinalysis did not show significant abnormality. Thyroid function tests were normal with T4 of 13.3 μg/dL and TSH of 4.09 mU/L. Serum glucose level was 5.1 mmol/L. Paraneoplastic markers such as anti-Yo, Ri, Ma, Hu, CV2, and amphiphysin were all negative. Magnetic resonance imaging (MRI) of brain showed cerebellar atrophy (Fig. 1). CT neck/thorax showed bilateral parotid and submandibular microcytic appearance with cystic lung and airspace opacities suggestive of lymphocytic interstitial pneumonia. Fine needle aspiration cytology (FNAC) of the left parotid gland was done and revealed reactive lymphoid hyperplasia.

With her background of poorly controlled PSS and clinical features of cerebellar signs consistent with the MRI findings, she was diagnosed with cerebellar degeneration secondary to PSS. She was treated with intravenous methylprednisolone 500 mg daily for 3 days and then maintenance therapy with oral prednisolone 30 mg daily. One gram of intravenous cyclophosphamide was also administered, and she was planned for further monthly cycles of cyclophosphamide. She was started on 200 mg of hydroxychloroquine. Tenofovir was given in view of her positive hepatitis B core antibody. She was seen by hematology team, and the team commented that the initial suspicious of marginal zone lymphoma in January 2018 was probably erroneous as the lesions did not progress. She received two cycles of monthly intravenous cyclophosphamide. Subsequently, she refused further treatment in our center and opted for a second opinion. The patient was lost to follow-up after that. Upon last follow-up, her neurological symptoms remained the same.

## Discussion

We report the case of a 37-year-old woman with primary Sjögren syndrome who presented with ataxia over 3 months associated with tremor of the limbs. Based on the presence of cerebellar signs with MRI brain findings, she was diagnosed with cerebellar degeneration secondary to PSS. Primary cerebellar degeneration is rarely associated with PSS. We evaluated possible causes of primary cerebellar degeneration in our patient, including alcohol, drug abuse, paraneoplastic manifestations, autoimmune disorders, and a rare manifestation of HIV infection [16]. Diagnosis by exclusion ultimately led to the conclusion that her cerebellar degeneration was due to poorly controlled PSS. Her disease was not well controlled since onset in 2014, which involved the joints, hematological, and neurological system.

Our patient had both peripheral and central nervous system involvement as evidenced by her previous history of mononeuritis multiplex and the current onset of cerebellar ataxia due to cerebellar atrophy. In contrast to the uniform features of the peripheral nervous system complications of PSS, CNS abnormalities are associated with a much wider spectrum of manifestations. In the largest cohort of 431 PSS patients published in 2010, the most frequent picture of CNS involvement was a recurrent subacute encephalopathy characterized by memory loss, cognitive dysfunction, visual disturbances, and dizziness [10]. The first description of cerebellar syndrome in association with PSS was defined by Attwood and Poser in 1961 [17]. Primary cerebellar degeneration is rarely associated with PSS. To our knowledge, there are only six case reports (Table 1) of cerebellar atrophy associated with PSS [11, 18–22].

**Table 1:** Previous reports of cerebellar degeneration secondary to PSS

	Age	Sex	Neurologic deficits	MRI brain findings	Autoantibody	Treatment received	Treatment effect
Terao *et al*. 1994	61	M	Ataxia, dysarthria, vertigo	Cerebellar and frontal lobe atrophy, multiple high-signal-intensity lesions	Antineuronal Ab (+)	Steroid	+
Owada *et al*. 2002	55	F	Motor weakness, ataxia, sensory disturbance, depression, apathy	Cerebellar and cerebral cortex atrophy	Antineuronal Ab (+)	Steroid	−
Kim *et al*. 2012	46	F	Gait and limb ataxia, depression, delusion	Cerebellar atrophy with an enlarged fourth ventricle and cisterna magna	Paraneoplastic Ab (−)	Steroid	−
Farhat *et al*. 2016	30	F	Cerebellar ataxia worsening over 4 years associated with tremor of the limbs and the head	Bilateral hyperintensities affecting periventricular white matter, with marked cerebellar atrophy	Paraneoplastic Ab (−)	Steroid and cyclophosphamide	−
Maciel *et al*. 2017	36	F	Ataxia, dysarthria	Cerebellar atrophy	Not done	Steroid and cyclophosphamide	−
Heidary *et al*. 2018	22	F	Ataxia with tremor of four limbs	Cerebellar atrophy	Not done	Steroid and cyclophosphamide	+

Ab, antibody; F, female; M, male; (+), positive; (−), negative

The first case of cerebellar ataxia with cerebellar atrophy was reported by Terao *et al*. in 1994 [18]. The patient was a 61-year-old gentleman who presented with progressive onset of ataxia and vertigo. His neurological symptoms improved with steroids alone. There was another study by Heidary *et al*. in 2018 in which the patient’s cerebellar ataxia improved with steroids and cyclophosphamide [19]. In contrast to these two studies, our patient did not have similar neurological improvement despite being given a high dose of steroid and cyclophosphamide. The suboptimal control of her disease since 2014 and delay in seeking medical treatment probably contributed to her poor outcome. Her cerebellar atrophy is most likely irreversible. In the other four case reports, there was no significant neurological improvement by medical treatment [11, 20–22]. To date, there is no consensus of a specific therapy for the management of Sjögren’s syndrome with CNS involvement.

The pathophysiology of cerebellar atrophy in SS has yet to be elucidated. Some studies have postulated that antibodies and ischemia may play a significant role, resulting in microinfarcts or macroinfarcts [4, 23]. In addition to cerebellar atrophy, Terao *et al*. [18] and Farhat *et al*. [21] reported additional signs of CNS inflammation, such as gadolinium enhancement or cerebellar white-matter fluid attenuated inversion recovery (FLAIR) hyperintensities, which were absent in our patient. The absence of hyperintense lesions on MRI suggests that vasculitis or demyelination is unlikely in our case. Owada *et al*. [11] and Terao *et al*. [18] reported presence of an autoantibody reactive to Purkinje cells in the CSF. We did not test for the presence of common antineuronal antibodies as the test was unavailable. However, most published cases of SS-related cerebellar degeneration reported negative antineuronal antibodies screen [20, 21]. The course of the disease of neuro-Sjögren can be multiple sclerosis-like with relapsing–remitting modality or progressive. In Massara’s paper, it was proven that CNS involvement may even precede clinical diagnosis of SS by many years, with patients misdiagnosed as multiple sclerosis fulfilling the diagnosis criteria [10].

The risk of B-cell lymphoma in PSS patients is 15 to 20 times higher than in the general population (lifetime risk, 5–10%), a finding that has been attributed to the chronic B-cell activation in this PSS. These lymphomas are mostly B-cell non-Hodgkin’s lymphomas with a predominance of the low-grade, marginal-zone histological type. Lymphomas often develop in organs in which PSS is active, such as the salivary glands and thus are primarily mucosa-associated lymphoid tissue (MALT) lymphomas [24, 25]. Our patient is at high risk of having lymphoma based on positive rheumatoid factor, parotid gland enlargement, and chronic uncontrolled disease. Close monitoring of her risk of lymphoma is warranted. The initial suspicion of marginal lymphoma in January 2018 was probably erroneous as the lesions did not progress.

## Conclusion

Our patient had severe cerebellar dysfunction that did not respond to medical treatment. Early diagnosis and aggressive treatment of cerebellar degeneration in PSS is essential to ensure good outcome. Further consensus on the treatment is needed.

## Data Availability

The clinical data and images in this article are available from the authors upon reasonable request.

## Abbreviations

PSS: Primary Sjögren syndrome
SLE: Systemic lupus erythematosus
CNS: Central nervous system
MRI: Magnetic resonance imaging
CT: Computed tomography
SS: Sjӧgren syndrome
BMAT: Bone marrow aspiration trephine
FNAC: Fine needle aspiration cytology
PET scan: Polyethylene terephthalate scan
CSF: Cerebral spinal fluid
MS: Multiple sclerosis
